# Uva-ursi extract and ibuprofen as alternative treatments of adult female urinary tract infection (ATAFUTI): study protocol for a randomised controlled trial

**DOI:** 10.1186/s13063-017-2145-7

**Published:** 2017-09-08

**Authors:** Jeanne Trill, Catherine Simpson, Frances Webley, Mike Radford, Louise Stanton, Tom Maishman, Angeliki Galanopoulou, Andrew Flower, Caroline Eyles, Merlin Willcox, Alastair Hay, Gareth Griffiths, Paul Little, George Lewith, Michael Moore

**Affiliations:** 1 Primary Care and Population Science, University of Southampton Faculty of Medicine, Aldermoor Health Centre, Southampton, SO16 5ST England; 2 Southampton Clinical Trials Unit, University of Southampton Clinical Trials Unit MP131, Southampton General Hospital, Tremona Road, Southampton, SO16 6YD England; 3 Centre for Academic Primary Care, National Institute for Health Research (NIHR) School for Primary Care Research, Population Health Sciences, Bristol Medical School, University of Bristol, Whatley Road, Bristol, BS8 2PS England

**Keywords:** Antibiotic resistance, Urinary tract infection, Uva-ursi, NSAID, Ibuprofen

## Abstract

**Background:**

Women with acute uncomplicated urine infection are usually treated with antibiotics. One trial has demonstrated that delayed antibiotic treatment offered without symptom relief results in a modest reduction in antibiotic use. There is some evidence that ibuprofen provides symptom relief and reduces antibiotic use. Uva-ursi, a herbal product, has a traditional use for urinary infection symptom relief. We set out to test: in adult women with suspected UTI who accept the delayed prescription strategy: Do NSAIDs or uva-ursi (a herbal product) provide relief from urinary symptoms and reduce antibiotic use.

**Methods/design:**

Adult women with suspected urinary tract infection presenting to primary care will be randomised using a factorial trial design in which patients will be randomised to one of two interventions as below:

- Group 1 – Uva-ursi + advice to take ibuprofen
- Group 2 – Placebo + advice to take ibuprofen
- Group 3 – Uva-ursi + no advice to take ibuprofen
- Group 4 – Placebo + no advice to take ibuprofen

Patients and physicians will be blinded to the randomised group for the herb.

The main outcome is symptom severity at days 2–4 recorded in a validated, self-report diary used in previous studies.

Secondary outcomes include antibiotic use and symptom duration.

In total the trial will require 328 patients in order to achieve at least 90% power for the primary endpoint and 80% for the secondary endpoint.

In accordance with CONSORT guidelines all comparative analyses will be conducted on an intention-to-treat basis using SPSS or similar package.

**Discussion:**

The outcomes from this trial have the potential to modify the current approach to the management of acute urinary symptoms with less dependence on the use of antibiotics.

**Trial registration:**

ISRCTN registry, ID: ISRCTN43397016. Registered on 11 February 2015.

**Electronic supplementary material:**

The online version of this article (doi:10.1186/s13063-017-2145-7) contains supplementary material, which is available to authorized users.

## Background

Urinary tract infections (UTIs) are one of the most common conditions seen in female patients in general practice for whom the lifetime risk is 50% and the annual incidence is estimated to be over 10% [1]. It accounts for 1–3% of all general practitioner (GP) consultations and the majority of women (93%) are prescribed antibiotics [2]. This strategy is poorly directed at women with proven infection [3], although women with dipstick-negative symptoms show evidence of shortened illness duration following empirical antibiotic treatment [4]. Urine is now the most commonly received specimen in microbiological laboratories, but more than 20% of isolates are resistant to trimethoprim and cephalosporins and 50% are resistant to amoxicillin [5]. Recent studies have documented a worse prognosis and higher treatment costs for women with UTI with antibiotic-resistant organisms [2, 6], and antibiotic resistance in urine isolates is linked to prior antibiotic exposure [7, 8]. However, limited data from trials suggest that uncomplicated UTIs have a good long-term prognosis, with low risk of complications [5, 9, 10]. Urinary symptoms usually settle 3–4 days after consultation but a shortened illness is experienced by women prescribed antibiotics in the absence of resistance [2, 11]. So, antibiotics do provide some relief from the distressing symptoms of UTI [12], but it is still unclear how best to target them or whether alternative symptomatic treatments might reduce reliance on antibiotics.

The delayed antibiotic prescription strategy results in a substantial reduction in antibiotic prescribing rates in respiratory illness and has gained credence as a rational approach to management endorsed by NICE [12, 13]. In respiratory illness it is considered to be a more effective way to modify consultation behaviour than patient education [14]. It has been shown to be acceptable to women with cystitis but with only a modest reduction in antibiotic use [15]. However, it is unlikely to be widely adopted without an alternative treatment to relieve the symptoms of infection. Two potential candidates have been identified to provide such relief from symptoms. A non-steroidal anti-inflammatory drug (NSAID) and a herbal product with traditional use in UTI in women which, if successful, could facilitate the adoption of the delayed strategy and further reduce antibiotic use in UTI.

## Background to alternative treatments

### NSAIDs

In one small study (*n* = 80) women with clinically suspected urinary infection were randomised to treatment with antibiotics or ibuprofen [16]. It was reported that ibuprofen provided similar levels of symptom relief to antibiotics (although this did not reach statistical significance in this small study) but, as would be expected, a larger proportion of women in the ibuprofen group required rescue antibiotics (33% versus 18%). A follow-up, fully powered trial has subsequently confirmed a substantial (66%) reduction in antibiotic use (94/241 ibuprofen versus 283/243 antibiotic) [17], although symptom burden was greater in the ibuprofen group: 70% symptom-free after 1 week with ibuprofen compared to 82% in the antibiotic group. This suggests that NSAIDs could reduce antibiotic use at the expense of poorer symptom relief.

### Herbal product

Herbal products for the relief of urinary symptoms are widely available over-the-counter (OTC) with a traditional-use licence, although they have never been subjected to rigorous efficacy studies. The leaf extract of *Arctostaphylos uva-ursi* (uva-ursi or bearberry) has been approved for use for urinary tract inflammation by the German Federal Institute for Drugs and Medical Devices and is available on prescription in Germany for this indication. It is reported to have diuretic, urinary antiseptic, astringent and anti-inflammatory properties [18]. The extract constituents include flavonoids, iridoids, hydroquinone glycosides (mainly arbutin), tannins and terpenoids. In-vitro studies have demonstrated antibacterial activity against a variety of organisms including *Escherichia coli*, the most prevalent urinary pathogen. The antimicrobial action has been attributed to the hydroquinone derivatives, especially arbutin [18].

Uva-ursi is widely available OTC in the UK and there is preliminary evidence suggesting that it provides symptom relief when used in acute UTI. One study of 309 women found that those recommended to use Uvacin (an OTC preparation including uva-ursi) experienced shorter illness duration, although the numbers who subsequently reported use of the product was low (14% with advice to use versus 1% with no advice) [15]. There is also some limited evidence that the prophylactic use of UVA-E, containing an aqueous/alcoholic extract of uva-ursi leaves and *Taraxacum offinale* (dandelion) root (*n* = 57) is effective for women with recurrent UTI [19].

### Main research question

The main study has two primary aims to assess the impact on UTI symptom severity of:

1. Uva-ursi compared to placebo, and
2. Advice to take ibuprofen compared to no such advice

Our secondary research objectives are to:

1. Examine the impact on the use of antibiotics and symptom duration
2. Describe the patient/practitioner barriers to the implementation of a delayed antibiotic prescription approach, and
3. Ascertain attitudes to the use of herbal medication

## Methods/design

### Study design and setting

ATAFUTI is a multicentre, factorial (2 × 2), randomised, double-blind, placebo-controlled trial of uva-ursi, and an open, pragmatic trial of advice/no advice to take ibuprofen. UK patients access in-hours care largely through family practices which hold registered patient lists; services are also available from walk-in facilities where no prior registration is needed and may be used for more acute symptoms. Out-of-hours services are provided at a district level from separately commissioned providers. Recruitment will be conducted in up to 60 GP family practices, walk-in centres and out-of-hours primary care practices across the South Midlands, East Anglia, the South, Southeast and Southwest England. See Additional file 1 for a completed Standard Protocol Items: Recommendations for Interventional Trials (SPIRIT) 2013 Checklist.

- Group 1 – Uva-ursi + advice to take ibuprofen
- Group 2 – Placebo + advice to take ibuprofen
- Group 3 – Uva-ursi + no advice to take ibuprofen
- Group 4 – No uva-ursi + no advice to take ibuprofen

The target population will be women presenting with symptoms of uncomplicated acute cystitis to primary care sites. All study centres will be selected from members of the National Institute for Health Research (NIHR) clinical research network related to three recruitment centres (Southampton, Bristol and Oxford) and who express an interest in the study.

Prior to participation all patients will be given a Participant Information Sheet (PIS) detailing the study protocol, and be required to give informed written consent (IC). Those agreeing and who are eligible to take part will need to provide a mid-stream urine specimen for confirmation of bacteriuria at baseline. Amongst women of child-bearing age a urine pregnancy test will also be performed. A subsample (20%) will be asked to provide a second urine sample after 4 days to be analysed for metabolites of uva-ursi (see Additional files 2, 3, 4 and 5 for the PIS and IC Forms for the main trial).

Patients will take the study medication for 3–5 days, and will complete a symptom diary for up to 2 weeks (see Additional file 6 for the diary). All participants will be issued a prescription for delayed antibiotics to be used if symptoms worsen, or after 3 to 5 days if symptoms fail to improve. A notes review will be undertaken at 3 months to document GP consultations regarding a recurrence of UTI.

### Participants’ eligibility

Patients meeting the following criteria may be included in the trial: female primary-care patients (aged 18–70 years) who, upon presenting to primary care with dysuria, urgency or frequency of urination, are suspected by a GP or nurse practitioner to have a lower UTI. They must be willing to accept a delayed prescription for antibiotics. Patients meeting any of the following exclusion criteria will be excluded from the study: known or suspected pregnancy; breastfeeding; suspected upper UTI (presenting with back pain, fever > 38 °C, systemic illness); who require immediate antibiotics; are within 7 days of taking antibiotics; frequent recurrent UTI (more than three UTI episodes in the past 12 months); known contraindications or cautions to ibuprofen; using an NSAID or taking an uva-ursi preparation and unwilling or unable to discontinue for the study period; diabetes; an immunodeficiency state and taking long-term corticosteroids or chemotherapy; bladder surgery including cystoscopy in the last 4 weeks; currently taking warfarin, or coagulopathy; recruited to another trial in the previous 4 weeks.

### Recruitment and randomisation

**Fig. 1:**
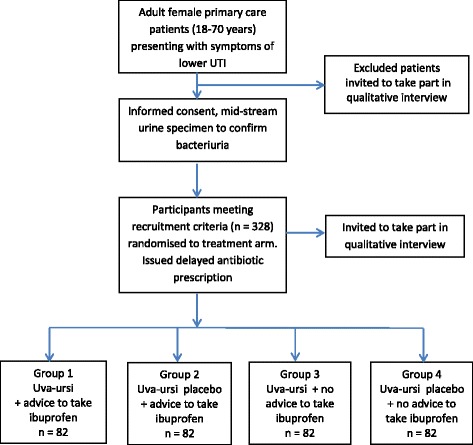
Recruitment and randomisation

Eligible participants will be identified and approached in primary care centres (Fig. 1). The patient’s consent to participate in the trial will be obtained prior to any trial-related procedures, which includes pregnancy testing and after a full explanation of the treatment options has been given. Consent will be taken by an appropriately trained research nurse or delegate. Patients who decline to participate in the main study, as well as participants who enter the main study, will be asked whether they consent to the storage of their contact details so that a qualitative researcher may contact them to invite them to participate in a qualitative interview about views on herbal treatment and potential barriers to study participation. The qualitative researcher will collect consent for conducting the interview immediately prior to commencement of the interview using the qualitative interview Consent Form.

Patients who are eligible for the study will be randomly assigned to one of the four treatment arms through allocation of the next available sequentially numbered ‘Patient Pack’, with participants and clinicians blinded to the uva-ursi groups. The Patient Pack number will determine their Participant ID. Advice leaflets supporting use of ibuprofen will be contained within treatment packs and not visible prior to randomisation. Recruiting practitioners will endorse ibuprofen use only when directed after opening the pack, no ibuprofen will be provided in the pack but patients randomised to the ibuprofen groups will be given a card stating the dosing regimen to be followed. The recruiting practitioners will be free to prescribe ibuprofen, but for participants paying prescription charges a less costly option would be to purchase a supply of ibuprofen from a pharmacy. No further contact with the participant by the recruiting physician will be specified, repeat consultations arising from clinical need will be collected by independent note review. Outcome assessors will also be blind to allocation of the herb but advice and prescription of ibuprofen will be available in the notes at the time of notes review. The randomisation concealment list will be produced centrally by the Southampton Clinical Trials Unit and allocation ratio for control to intervention arms will be 1:1.

### Study interventions

The Investigational Medicinal Product (IMP) will comprise uva-ursi extract containing 20% arbutin supplied by Temmler Pharma GmbH & Co (Germany), encapsulated to Good Manufacturing Practice (GMP) and IMP standards by Essential Nutrition Limited (UK). To maintain blinding, the matching placebo will contain sugar beet fibre (Fibrex®), an inert substance with a similar colour and a herbal flavour, from Nordic Sugar (Denmark). The trial medication will be stored and dispensed centrally in accordance with Good Clinical Practice (GCP), and supplied in a medication securitainer contained in a Patient Pack with instructions for the GP to give advice or not to take ibuprofen.

The daily dose of uva-ursi, which has been submitted to, and approved by, the Medicines and Healthcare Regulatory Committee (MHRA), will be 3600 mg (3 × 400-mg capsules) to be taken orally three times a day thus providing a total of 686 mg arbutin. Participants will be asked to take the study medication for 3 days and up to 5 days. A prescription for rescue antibiotics will be made available for all participants with instructions to take if adequate symptom relief is not obtained from the study medication.

A structured Advice Sheet and a card detailing the ibuprofen dose and approved medication will be provided to participants randomised to the ibuprofen arm. A daily dose of 1200 mg, to match the dose used in the previous trials [16, 17], will be recommended or prescribed on request. The group not randomised to the ibuprofen arm will not receive any advice on this drug. The intervention is advice only and falls outside regulation by the MHRA.

### Assessment and follow-up

Participants will be required to maintain a daily symptom diary used in previous studies of UTI [20, 21], grading severity of their presenting and subsequent UTI symptom(s). To assist completion they will be contacted by a research assistant after 3 days. No questions will be asked about compliance or delayed prescription. Completed diaries will be returned to the Southampton Centre in a freepost envelope. In the event of non-return after 3 weeks participants will be contacted to prompt diary return or complete a brief symptom inventory using recall if the diary is mislaid.

Symptom severity for the primary outcome will be recorded in the symptom diary where day 1 is completed on the day of the consultation. Previous factor analysis of the diary has shown that symptoms can be grouped into two factors, ‘frequency’ and ‘unwell’ symptoms [2]. The primary outcome consists of the mean symptom score of the ‘frequency factor’ items on days 2–4 following consultation on day 1.

Primary outcome:

- Symptom severity on days 2–4 using validated diary data [2, 15, 22].

Secondary outcomes:

- Use of antibiotics
- Individual data from each of the four groups for primary outcome and use of antibiotics
- Duration of moderately bad symptoms
- Duration of symptoms until little or no problem
- Total symptom burden derived from diary data
- Re-consultation in 1 month with UTI from notes review
- Re-consultation in 3 months with UTI from notes review

Exploratory analysis:

- Differential effects on primary outcome depending on culture results

Adverse events will be recorded as detailed below under safety considerations.

**Fig. 2:**
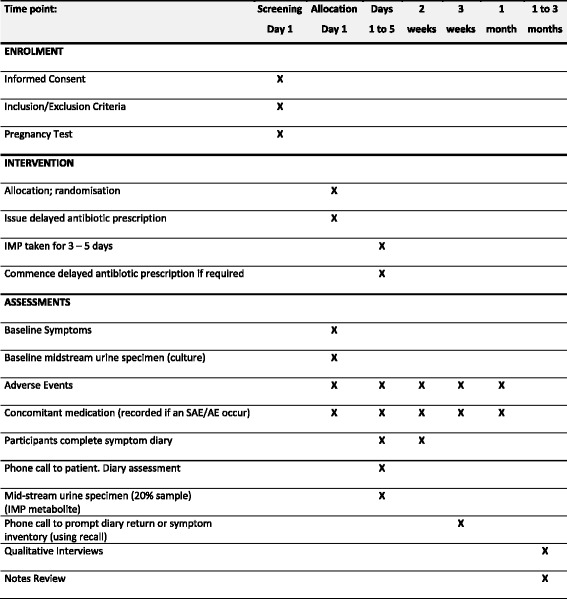
Standard Protocol Items: Recommendations for Interventional Trials (SPIRIT) Figure showing schedule of procedures (enrolment, intervention and assessments)

Data and all appropriate documentation will be stored for a minimum of 5 years after the completion of the trial, including the follow-up period. The Standard Protocol Items: Recommendations for Interventional Trials (SPIRIT) Figure showing the schedule for enrolment, interventions and assessments is provided in Fig. 2.

### Qualitative interviews

Attitudes towards the delayed prescribing of antibiotics in favour of treating the symptoms with ibuprofen and/or herbal medicine will be explored in a nested qualitative study. In-depth semistructured telephone interviews will be conducted amongst 20–30 women who have taken part in the trial or who were approached but not randomised, and amongst 10–15 GPs from the same GP practices. Participants will be given an invitation letter, a PIS and a Consent Form to be signed and returned to the researcher, who will contact them about a month, but no more than 3 months, after taking part (see Additional files 7, 8, 9 and 10 for the PIS and IC Forms). Interviews will be conducted using an interview schedule, will be 15–30 min in duration and will be recorded and transcribed verbatim. All interviews will be analysed using thematic analysis [23], facilitated by NVivo software.

### Safety considerations and withdrawal

The discontinuation of study medication will occur if there is evidence of progressive infection or upper urinary tract symptoms which warrant immediate antibiotic therapy, pregnancy, any development of toxicity, or a concurrent illness in which the investigator’s opinion precludes further treatment. Participants are all provided with a prescription for rescue medication and are able to self-determine when to collect the prescription depending on symptom burden and whether to continue or stop the study medication(s).

Most adverse events (AEs) and adverse drug reactions that occur in this trial, whether serious or not, are anticipated to be expected treatment-related toxicities due to the trial medication. The assignment of the causality will be made by the primary care physician responsible for the participant. A pre-existing condition will not be reported as an AE unless the condition worsens by at least one Common Terminology Criteria for Adverse Events grade during the trial.

A major concern over the withholding of antibiotics is the potential for progressive infection and upper UTI. There are, however, limited data regarding the absolute risk of upper tract infection following lower tract infection in women. In a meta-analysis of placebo versus antibiotic trials, antibiotics were more effective than placebo but associated with more adverse effects [11]. In the two trials that reported the incidence of pyelonephritis, the range in those treated with antibiotics was 0–0.15% and in those treated with placebo 0.4–2.6% (not significant). In the recent trial of ibuprofen versus antibiotic [17] the number of episodes of pyelonephritis was five in the placebo arm compared to one in the antibiotic arm although none required hospital admission. A similar trend was noted for worsening symptoms and febrile UTIs. Episodes of suspected upper UTI will be reported and all AEs will be recorded from the time that the patients signs the Consent Form until 4 weeks after randomisation.

Notification of adverse events will be through one of three mechanisms: (1) all participants will carry a trial card with relevant telephone numbers to call should an AE occur, (2) GP practices will notify the study team of any AE reported to the practice and (3) AE information will be collected at notes review after 3 months.

Due to the low risk of the IMP treatments and that any emergency clinical decisions would be unaffected by knowledge of the treatment group, a normal-working-hours unblinding service will be provided.

Participants are also free to withdraw at any time without giving a reason.

All AEs that may be related to the study will be recorded on the Adverse Events Form and sent to the SCTU within 1 month of the form being due. As adults on average see their GP approximately five times per year for a variety of routine and unscheduled appointments (e.g. for medication review, self-limiting minor illnesses and long-term conditions unrelated to urinary infection), many medical encounters are of no relevance to the study. Events that will be reported include any judged by the principal investigator at the site to be possibly related to the study. In particular, all medical encounters related to the following medical areas or symptoms will be recorded in the Adverse Events Form:

- Abdominal symptoms: any events relating to abdominal discomfort or other symptoms
- Urine infection: any events relating to UTI
- Medication: any events relating to study medication

The practitioners providing care for the patient are advised to record any event for which there is uncertainty as to whether it is study-related or not, and to discuss with the local principal investigator (PI) or chief investigator (CI).

Fatal or life-threatening serious adverse events (SAEs) and suspected unexpected serious adverse reactions (SUSARs) will be reported within 24 h of the local site becoming aware of the event. The SAE/SUSAR Form asks for nature of event, date of onset, severity, corrective therapies given, outcome and causality (i.e. unrelated, unlikely, possible, probably, definitely) as per standard reporting procedures.

### Power calculation and statistical analysis

In our previous study, the severity of frequency symptoms at 2–4 days was 2.15 (SD 1.18) in the immediate antibiotic group and 2.11 (mean difference −0.04, CI −0.47 to 0.40) in the delayed antibiotic group [15]. The clinically significant change in symptom severity based on previous consensus [21] and the previous UTI study would be a difference of 1 severity point in every two women, that is a mean severity difference of 0.5. In order to estimate the required sample size a power calculation was carried out using NQuery 3.0 with a two-sided significance level of 5% (alpha = 0.05) and power of 90% (beta = 0.1) based on a two-group *t* test of equal means. For the 2 × 2 factorial design, to detect a mean severity difference of 0.5 with SD 1.18 requires a sample of 60 per group, or 75 per group, so 300 in total allowing for 20% loss to follow-up.

An important secondary outcome is the proportion of participants who use antibiotics. In our previous study we achieved a reduction from 90% in the immediate antibiotic group to 77% 41/53 in the delayed group (without additional symptom relief). Thus, in this study we would anticipate the ‘placebo/no advice to take ibuprofen arm’ to experience 77% uptake of antibiotic. In a study comparing NSAID to antibiotic for UTI, only 33% of the NSAID group required rescue antibiotics so potentially this intervention may reduce antibiotic uptake to as little as 30%. A clinically significant reduction in antibiotic use would be 17% (i.e. from 77% to 60%) and would require a total sample of 65.5 per group or 262 in total, 328 allowing for 20% loss to follow-up (82 per group) for 80% power with a two-sided significance level of 5%, based on a chi-squared test of equal proportions.

In order to achieve at least 90% power for the primary endpoint and allowing 20% loss to follow-up, the trial requires four groups of 82 patients. If the loss to follow-up is higher than anticipated and increases to 30%, a total of 376 patients (94 per group) will be required. The trial is not powered for any interaction between factorial groups.

### Statistical analysis

All analyses will be performed on a full intention-to-treat basis, that is, all patients randomised will be included, and all patients will be analysed according to their allocated group whatever treatment they receive.

For the primary analyses, descriptive statistics will be obtained for the randomisation groups to characterise recruited patients and assess baseline comparability. The study will be reported in accordance with Consolidated Standards of Reporting Trials (CONSORT) guidelines [22].

For the primary outcome, analysis of covariance will be used to analyse symptom-severity data with imputation of missing data. Logistic regression will be used to analyse the dichotomous outcomes (antibiotic use, re-consultation in 1 month with UTI and re-consultation with UTI from notes review in the next 12 months). Negative binomial regression will be used to analyse duration of moderately bad symptoms. All analyses will be adjusted for the factorial design and, if necessary, for potential confounding variables, e.g. use of antibiotics.

For each outcome the following pairwise comparisons will be examined by calculating a 95% CI for the difference/odds ratio/incidence rate ratio:

1. Uva-ursi versus no uva-ursi: group 1 + group 3 patients versus group 2 + group 4 patients
2. Advice to take ibuprofen versus no advice to take ibuprofen: group 1 + group 2 patients versus group 3 + group 4 patients

In a previous observational cohort, the exploratory factor analysis of the severity of symptoms demonstrated two groups of symptoms: these were increased day frequency, increased night frequency and urgency and dysuria (a ‘frequency’ group of symptoms; Cronbach’s alpha = 0.77); and abdominal pain, restricted activities and feeling unwell (‘unwell’ group of symptoms; Cronbach’s alpha = 0.80). We will explore the effect of the interventions on symptoms in the frequency and unwell clusters.

The Data Monitoring Committee will review safety and efficacy data at least annually. Data management is directed by the standard operating procedures of the Southampton University CTU.

The main analysis will be carried out when all patients have completed the study. A statistical analysis plan will be written before the data are analysed.

## Discussion

The ATAFUTI study is the first double-blind, placebo-controlled, factorial randomised trial to investigate a traditional herbal medicinal product as an alternative treatment for UTIs in women, with the aim of reducing symptoms and reducing antibiotic consumption in primary care. The open, pragmatic arm of the trial also builds on existing research into symptom relief of uncomplicated UTI with ibuprofen, whereby two thirds of women (*n* = 248) with mild to moderate symptoms recovered without the need for antibiotics [17]. It will provide clinical evidence on the efficacy and safety of using ibuprofen and a traditional herbal medicine uva-ursi, either singularly or in combination for the relief of the distressing symptoms of UTI in women, one of the most common reasons for antibiotic prescription. The NSAID has known anti-inflammatory properties, and uva-ursi has demonstrated antibacterial properties against *E. coli* both in vitro as well as in urine samples of healthy volunteers [18].

The diary format has been previously validated and shown to be sensitive to change for other acute infections [22, 24]. By using this method it will allow the impact of the intervention on symptom severity and on the duration of symptoms to be investigated.

Should the main outcome of the trial be positive, the qualitative element of the study will provide insights into any reservations that patients and practitioners may have about accepting a recommendation/prescription for ibuprofen and/or uva-ursi, with delayed prescribing of antibiotics, which in turn will inform implementation of the findings. The finding will be disseminated by publication and presentation at conferences. Summary findings will be provided to participating practices.

## Trial status

Recruitment commenced in August 2015 and is due to complete by October 2016.

The study has been reviewed by the NHS Health Research Authority: South Central Hampshire A 14/SC/1143.

## Additional files

Additional file 1: SPIRIT_ATAFUTI (Checklist). (DOC 121 kb)

Additional file 2: Participant Information Sheet Main Trial. (PDF 218 kb)

Additional file 3: Participant Informed Consent Main Trial. (PDF 514 kb)

Additional file 4: Participant Information Sheet + day-4 urine sample. (PDF 218 kb)

Additional file 5: Participant Informed Consent + day-4 urine sample. (PDF 521 kb)

Additional file 6: Participant diary. (PDF 515 kb)

Additional file 7: Qualitative Research Patient Information Sheet. (PDF 195 kb)

Additional file 8: Qualitative Research Patient Informed Consent. (PDF 369 kb)

Additional file 9: Qualitative Research GP Information Sheet. (PDF 250 kb)

Additional file 10: Qualitative Research GP Informed Consent. (PDF 310 kb)

Additional file 11: Trial amendments. (PDF 114 kb)

Additional file 12: CSP NHS permissions for ATAFUTI. (PDF 107 kb)

## Abbreviations

AE: Adverse event
CONSORT: Consolidated Standards of Reporting Trials
GMP: Good Manufacturing Practice
GP: General practitioner
IMP: Investigational Medicinal Product
MHRA: Medicines and Healthcare Regulatory Committee
NSAID: Non-steroidal anti-inflammatory drug
OTC: Over-the-counter
PIS: Participant Information Sheet
UTI: Urinary tract infection
Uva-ursi: *Arctostaphylos uva-ursi*

## References

[1] Foxman B (2002). Epidemiology of urinary tract infections: incidence, morbidity, and economic costs. Am J Med.

[2] Little P, Merriman R, Turner S, Rumsby K, Warner G, Lowes J (2010). Presentation, pattern, and natural course of severe symptoms, and role of antibiotics and antibiotic resistance among patients presenting with suspected uncomplicated urinary tract infection in primary care: observational study. BMJ.

[3] O’Brien K, Hillier S, Simpson S, Hood K, Butler C (2007). An observational study of empirical antibiotics for adult women with uncomplicated UTI in general practice. J Antimicrob Chemother.

[4] Richards D, Toop L, Chambers S, Fletcher L (2005). Response to antibiotics of women with symptoms of urinary tract infection but negative dipstick urine test results double blind randomised controlled trial. BMJ.

[5] Christiaens TC, De MM, Verschraegen G, Peersman W, Heytens S, De Maeseneer JM (2002). Randomised controlled trial of nitrofurantoin versus placebo in the treatment of uncomplicated urinary tract infection in adult women. Br J Gen Pract.

[6] Butler CC, Hillier S, Roberts Z, Dunstan F, Howard A, Palmer S (2006). Antibiotic-resistant infections in primary care are symptomatic for longer and increase workload: outcomes for patients with *E. coli* UTIs. Br J Gen Pract.

[7] Hillier S, Roberts Z, Dunstan F, Butler C, Howard A, Palmer S (2007). Prior antibiotics and risk of antibiotic-resistant community-acquired urinary tract infection: a case-control study. J Antimicrob Chemother.

[8] Costelloe C, Metcalfe C, Lovering A, Mant D, Hay AD (2010). Effect of antibiotic prescribing in primary care on antimicrobial resistance in individual patients: systematic review and meta-analysis. BMJ.

[9] Nickel JC, Lee JC, Grantmyre JE, Polygenis D (2005). Natural history of urinary tract infection in a primary care environment in Canada. Can J Urol.

[10] Foxman B (2014). Urinary tract infection syndromes: occurrence, recurrence, bacteriology, risk factors, and disease burden. Infect Dis Clin North Am.

[11] Falagas ME, Kotsantis IK, Vouloumanou EK, Rafailidis PI (2009). Antibiotics versus placebo in the treatment of women with uncomplicated cystitis: a meta-analysis of randomized controlled trials. J Infect.

[12] Arroll B, Kenealy T, Goodyear-Smith F, Kerse N (2003). Delayed prescriptions. BMJ.

[13] NICE (2011). Prescribing of antibiotics for self-limiting respiratory tract infections in adults and children in primary care.

[14] Thoolen B, de Ridder D, van Lensvelt-Mulders G (2012). Patient-oriented interventions to improve antibiotic prescribing practices in respiratory tract infections: a meta-analysis. Health Psychol Rev.

[15] Little P, Moore MV, Turner S, Rumsby K, Warner G, Lowes JA (2010). Effectiveness of five different approaches in management of urinary tract infection: randomised controlled trial. BMJ.

[16] Bleidorm J, Gagyor I, Kochen M, Wegscheider K, Hummers-Pradie E (2010). Symptomatic treatment (ibuprofen) or antibiotics (ciprofloxacin) for uncomplicated urinary tract infection?—Results of a randomized controlled pilot trial. BMC Med.

[17] Gagyor I, Bleidorn J, Kochen MM, Schmiemann G, Wegscheider K, Hummers-Pradier E (2015). Ibuprofen versus fosfomycin for uncomplicated urinary tract infection in women: randomised controlled trial. BMJ.

[18] EMA European Medicines Agency (2012). Assessment report on *Arctostaphylos uva-ursi* (l.) Spreng. folium.

[19] Larsson B, Jonasson A, Fianu S (1993). Prophylactic effect of UVA-E in women with recurrent cystitis: a preliminary report. Curr Ther Res.

[20] Little P, Turner S, Rumsby K, Warner G, Moore M, Lowes J (2009). Dipsticks and diagnostic algorithms in urinary tract infection: development and validation, randomised trial, economic analysis, observational cohort and qualitative study. Health Technol Assess.

[21] Bates J, Thomas-Jones E, Pickles T, Kirby N, Gal M, Bongard E (2014). Point of care testing for urinary tract infection in primary care (POETIC): protocol for a randomised controlled trial of the clinical and cost effectiveness of FLEXICULT informed management of uncomplicated UTI in primary care. BMC Fam Pract.

[22] Watson L, Little P, Moore M, Warner G, Williamson I (2001). Validation study of a diary for use in acute lower respiratory tract infection. Fam Pract.

[23] Braun VC V (2006). Using thematic analysis in psychology. Qual Res Psychol.

[24] Little P, Rumsby K, Kelly J, Watson L, Moore M, Warner G (2005). Information leaflet and antibiotic prescribing strategies for acute lower respiratory tract infection—A randomized controlled trial. JAMA.

