# Supplementary material for: Uva-ursi extract and ibuprofen as alternative treatments of adult female urinary tract infection (ATAFUTI): study protocol for a randomised controlled trial
Source: Trials. 2017 Sep 8;18:421. doi: 10.1186/s13063-017-2145-7 (PMC5591533; doi:10.1186/s13063-017-2145-7)
Supplement: Supplementary file 2 — Participant Information Sheet Main Trial. (PDF 218 kb) [file 13063_2017_2145_MOESM2_ESM.pdf]

UNIVERSITY OF OXFORD
NUFFIELD DEPARTMENT OF PRIMARY CARE HEALTH SCIENCES
University of BRISTOL
UNIVERSITY OF Southampton

Participant Information Sheet
Main Trial

ATAFUTI
A Trial Investigating Alternative Treatments for Adult Female Urinary Tract Infection

Version number v8 22-04-16

Ethics number: 14/SC/1143

We would like to invite you to take part in a research study. Before you decide, it is important that you understand why the research is being carried out and what it will involve for you. Please take time to read the following information carefully and ask your GP or nurse if there is anything that is not clear or if you would like more information.

Part 1

What is the purpose of the study?
More and more infections are becoming resistant to antibiotics. Many urinary infections are caused by bacteria which are resistant to common antibiotics. The more we use antibiotics, the more resistant the bacteria will become. So it is important that we research alternative ways of treating urine infections.

This study will look to see if the herb 'Uva ursi' provides relief from symptoms in women with suspected Urinary Tract Infection (UTI). We want to know if this medicine can reduce symptoms and therefore reduce the need for antibiotic use. In addition, we are also interested to know whether advice from your GP or nurse to take another over the counter (OTC) treatment (one that does not require a prescription) to relieve your symptoms could also help reduce the use of antibiotics.

What are the treatments being tested?
This study is looking at a herb called Uva ursi (Bearberry) which has been used as a traditional treatment for urine infections. Some laboratory experiments suggest that it may be beneficial for treating UTI, but there have never been any proper trials in humans. We are studying the effectiveness of taking Uva ursi for relieving UTI symptoms. In addition we will be looking into whether advice from your GP or nurse to take another OTC treatment for the symptoms of your UTI will also be effective.

Why have I been invited?
You have been invited because you have a suspected UTI. We aim to recruit 328 women with UTIs into the study.

Do I have to take part?
No. It is up to you to decide whether or not to take part in the trial. If you decide not to take part, this will not affect the standard of care you receive.

What will happen to me if I agree to take part?
After carefully reading this information sheet and asking your nurse or doctor any questions you may have about the trial, you will be asked if you agree to take part in the trial. The nurse or doctor will then check that you are eligible to enter the study by asking questions about your medical history, any medication you are taking and **whether you are willing to accept a delayed prescription for antibiotics**. You will be asked to sign a consent form. The consent form will confirm that you have read and understood the information in this document and that you give your permission to take part in the trial.

<u>Questions and tests</u>
You will be asked questions about your UTI symptoms. You will also be asked to provide a urine specimen that will be sent for laboratory analysis to confirm a urinary infection is present. A pregnancy test will also be performed to confirm that you are not pregnant before entering the trial.

If you were to become pregnant whilst taking the study medication you will be followed up by the research staff at your GP surgery until the baby is born. This is standard practice for all research.

<u>Allocation to a treatment group</u>
Sometimes we don't know which way of treating people is best. To find out we need to compare different treatments. We put people into groups and give each group a different treatment. The results are compared to see if one is better. To try to make sure the groups are the same to start with, each participant is put into a group by chance (randomly).

In this trial you will be allocated to receive either ‘Uva ursi’ 1200mg three times a day or placebo and you will also have a 50% chance of being recommended to take an over the counter treatment.

This means there is a 1 in 4 chance of receiving no symptom relief treatment.

You will not know whether you have been given the real or placebo herb ‘Uva ursi’ and neither will your nurse or doctor. However, you will be provided with a trial card which details the medication involved in the trial and contact numbers so that, in the unlikely event of a medical emergency, it is possible to find out whether you have been taking the real or placebo herb ‘Uva ursi’.

<u>Trial Medication</u>
You will be issued with a five day supply of the trial medication and be asked to take this for three days or up to five days if you still require help with your symptoms. If you are in the group receiving advice to take another OTC treatment you will be given a card with details of the treatment and again you will be asked to take the OTC treatment for three days or up to five days.

<u>Antibiotic prescription</u>
Along with your trial medication you will be given an antibiotic prescription by your GP or nurse practitioner that can be used if needed, for example if your symptoms are not improved with study medication, or significantly worsen. **You are asked, if at all possible, to wait for three days before using the antibiotic prescription.**

There are no anticipated interactions between the trial medication and antibiotics. If your symptoms persist and you decide to take the antibiotics then you can decide on whether to continue or stop the trial medication

Symptom Diary

You will be given a two week paper diary to record your UTI symptoms and all treatments that you have taken for your UTI, including the trial medication. You will be asked to complete the diary every day for up to 14 days or until your symptoms have settled. We will ask you to return the completed diary to the Southampton Clinical Trials Unit in the Freepost envelope provided.

On or shortly after the third day that you have been taking the study medication you will receive a phone call from a member of the research team to answer any questions you may have about completing the diary. In order for the research team to be able to contact you we will need to keep a record of your contact details. Your details will be stored securely at the Southampton Clinical Trials Unit in a locked filing cabinet in a restricted locked environment and/or electronically with password protection. This personal data will not be linked to any other trial related documents and will only be accessible to and used by designated members of staff following strict guidelines.

Once you have returned your diary your participation in the trial is complete unless you subsequently agree to be interviewed or take part in a group discussion about alternative treatments for UTIs. You will be given a letter and an information sheet explaining what is involved in this part of the trial by your GP or practice nurse. You will be able to take this home with you so that you can decide whether you wish to take part.

The information that you record in the diary is very important to the success of the trial. If after three weeks, we have not received your diary or if there is information missing a member of the research team will call you to try to collect this key information.

What are the side effects of any treatment received when taking part?

Side effects of Uva ursi are uncommon and usually mild. However, in much larger doses than used in this trial Uva ursi may cause nausea, vomiting and upset stomach, and urine may darken.

The capsules may interfere with the absorption of some vitamins and should be taken separately from any iron or vitamin supplements.

The most common side effect of the OTC treatment is stomach pain, and occasionally stomach ulcers. Some patients with asthma also experience a worsening of their asthma. Therefore if you have had stomach ulcers or asthma you should not take part in this study. Your GP will know whether you have had any other side-effects from the OTC treatment and whether you should participate in the trial.

What are the other possible disadvantages and risks of taking part?

It is possible that the uncomfortable symptoms of your Urinary Tract Infection may last longer. A prescription for antibiotics will be available should you wish to start taking them. Very rarely untreated urinary infection can spread to the kidneys. If this happens you would become more unwell and develop back pain, high fever and vomiting. A kidney infection needs urgent treatment and you may need a longer course of antibiotics. You must contact your doctor if any of these symptoms develop.

What are the possible benefits of taking part?
It is not known whether you will have any personal benefit from taking part in this study. However your participation will help to give important information about how best to treat people with UTI in the future.

This completes Part 1 of the Information Sheet.
If the information in Part 1 has interested you and you are considering participation, please read the additional information in Part 2 before making any decision.

Part 2

What will happen if I don't want to carry on with the study?
You can withdraw from the study at any time and you don't have to give a reason. The standard of your care will not be affected. Information collected up to the time you withdraw may still be used.

What if there is a problem?
If you have a concern about any aspect of this study, you should ask to speak with the researchers who will do their best to answer your questions (Tel: 023 8120 5171 to speak to the Trial Coordinator. Additional contact details are given under 'Further Information' below). Alternatively you can contact Research Governance Manager: Research Governance Office, Corporate Services, Building 37, Room 4079, University of Southampton, University Road, Highfield, Southampton, SO17 1BJ, Tel: 023 80 595058

In the unlikely event that something does go wrong and you are harmed during the research and this is due to someone's negligence then you may have grounds for a legal action for compensation against the University of Southampton but you may have to pay your legal costs. If you remain unhappy and wish to complain formally, you can do this through the normal National Health Service complaints Procedure. Details can be obtained through the following NHS web site.
http://www.nhs.uk/choiceintheNHS/Rightsandpledges/complaints/Pages/AboutNHScomplaints.aspx

During the study, if there is an emergency please contact your GP.

Will my taking part in this study be kept confidential?
If you join the study, some parts of your medical records and the data collected for the study will be looked at by authorised persons from the University of Oxford, University of Bristol and the Southampton Clinical Trials Unit (SCTU) (who have organised the research). They may also be looked at by representatives of regulatory authorities to check that the study is being carried out correctly. All information which is collected about you during the course of the research will be kept strictly confidential.

If you are recruited from a walk in centre, we will ask for your consent to notify your GP of your participation in this study.

What will happen to the results of the research study?
The findings from this study will be published after the study data has been analysed (approximately during 2016). No identifiable information will be included which could compromise the confidentiality of the study participants. Any participant who wishes to obtain a copy of the publication or a summary of the results of the trial should contact the Southampton Clinical Trials Unit, University of Southampton MP131, Southampton General Hospital, Tremona Road, Southampton, Hants, SO16 6YD.

Results will be analysed by the researchers and published in medical journals. If results are conclusive they may be used to influence future NHS guidelines for treatment of Urinary Tract Infection.

Who is organising and funding the research?
This study has been organised by the Universities of Southampton, Oxford and Bristol and the study is being run by the Southampton Clinical Trials Unit. The study is funded by a grant from the National Institute for Health Research, National School for Primary Care Research. The study Sponsor is the University of Southampton.

Who has reviewed the study?
All research in the NHS is looked at by an independent group of people, called a Research Ethics Committee, to protect your interests. This study has been reviewed by NRES Committee South Central – Hampshire A.

Further Information and contact details
Please contact: Trial Co-ordinator, Catherine Simpson at the Southampton Clinical Trials Unit, if you have any questions.

Tel: 023 8120 5171
Email: CTU@soton.ac.uk

If you have any further questions about your illness or this study, please discuss them with your GP.

For further information about Urinary Tract Infection you may also find it helpful to look at the following NHS internet link:
http://www.nhs.uk/conditions/Urinary-tract-infection-adults/Pages/Introduction.aspx

Thank you for taking the time to read this information sheet.
