# Supplementary material for: Uva-ursi extract and ibuprofen as alternative treatments of adult female urinary tract infection (ATAFUTI): study protocol for a randomised controlled trial
Source: Trials. 2017 Sep 8;18:421. doi: 10.1186/s13063-017-2145-7 (PMC5591533; doi:10.1186/s13063-017-2145-7)
Supplement: Supplementary file 3 — Participant Informed Consent Main Trial. (PDF 514 kb) [file 13063_2017_2145_MOESM3_ESM.pdf]

UNIVERSITY OF Southampton

**Ethics number:**	**14/SC/1143**
**Centre ID:**	
**Participant's Initials:**	**(If no middle initial insert '-')**
**Participant's Month & Year of Birth:**	M M M Y Y Y Y
**Participant Trial ID:**	

ATAFUTI

A Trial to Investigate Alternative Treatments of Adult Female Urinary Tract Infection.

CONSENT FORM – Main Trial

Name of Researcher: Professor Michael Moore

Please Initial Box

1. I confirm that I have read and understand the participant information sheet (version xx dated xx/xx/xx) for the above trial. I have had the opportunity to consider the information, ask questions and have had these answered satisfactorily.

2. I understand that my participation is voluntary and that I am free to withdraw at any time without giving any reason, and without my medical care or legal rights being affected.

3. I understand that I will be asked to delay taking my treatment of antibiotics.

4. I understand that I have a 1 in 4 chance of receiving no symptom relief treatment.

5. I consent to the collection and use of information about me in accordance with the participant information sheet.

UKCRC Registered Clinical Trials Units

NHS National Institute for Health Research

6. I consent to having a pregnancy test if required. ☐

7. I am willing to provide a urine sample for laboratory analysis to confirm a urinary infection is present. ☐

8. I understand that relevant sections of my medical notes and data collected during the trial may be looked at by responsible individuals from the Sponsor, University of Southampton or Sponsor delegates from the Southampton Clinical Trials Unit, the University of Bristol & the University of Oxford or by regulatory authorities where it is relevant to my taking part in research. I give permission for these individuals to have access to my records. ☐

9. I understand that any data collected from me up to the time of my withdrawal may continue to be used in the above study. ☐

10. I agree for my GP to be informed of my involvement in this trial. ☐

11. I agree to take part in the above study. ☐

<u>Optional</u>

12. I am willing to be contacted by a research assistant to discuss completion of the diary as explained in the participant information sheet. ☐

For the purposes of the Data Protection Act 1998 the data controller is the University of Southampton and any inquiries relating to your personal information may be addressed to the Southampton Clinical Trials Unit, MP131, Southampton General Hospital, Southampton, SO16 6YD.

______________________	______________________	______________________
Name of Patient	Signature	Date
______________________	______________________	______________________
Name of Person taking consent	Signature	Date

When completed:
1 (original) signed consent form to be kept in researcher site file
1 copy for the participant
1 copy to be kept in Medical Notes
