# Supplementary material for: Uva-ursi extract and ibuprofen as alternative treatments of adult female urinary tract infection (ATAFUTI): study protocol for a randomised controlled trial
Source: Trials. 2017 Sep 8;18:421. doi: 10.1186/s13063-017-2145-7 (PMC5591533; doi:10.1186/s13063-017-2145-7)
Supplement: Supplementary file 5 — Participant Informed Consent + day-4 urine sample. (PDF 521 kb) [file 13063_2017_2145_MOESM5_ESM.pdf]

Ethics number: 14/SC/1143

Centre ID: ☐☐☐

Participant's Initials: ☐☐☐ (If no middle initial insert '-')

Participant's Month & Year of Birth: M M M Y Y Y Y

Participant Trial ID: ☐☐☐☐☐☐

ATAFUTI

A Trial Investigating Alternative Treatments of Adult Female Urinary Tract Infection.

CONSENT FORM

<u>Main Trial + Day 4 Urine Collection</u>

Name of Researcher: Professor Michael Moore

Please Initial Box

1. I confirm that I have read and understand the participant information sheet (version xx dated xx/xx/xx) for the above trial. I have had the opportunity to consider the information, ask questions and have had these answered satisfactorily. ☐
2. I understand that my participation is voluntary and that I am free to withdraw at any time without giving any reason, and without my medical care or legal rights being affected. ☐
3. I understand that I will be asked to delay taking my treatment of antibiotics. ☐
4. I understand that I have a 1 in 4 chance of receiving no symptom relief treatment. ☐
5. I consent to the collection and use of information about me in accordance with the participant information sheet. ☐
6. I consent to having a pregnancy test if required. ☐

7. I am willing to provide a urine sample for laboratory analysis to confirm a urinary infection is present. ☐

8. I understand that relevant sections of my medical notes and data collected during the trial may be looked at by responsible individuals from the Sponsor, University of Southampton or Sponsor delegates from the Southampton Clinical Trials Unit, the University of Bristol & the University of Oxford or by regulatory authorities where it is relevant to my taking part in research. I give permission for these individuals to have access to my records. ☐

9. I understand that any data collected from me up to the time of my withdrawal may continue to be used in the above study ☐

10. I agree for my GP to be informed of my involvement in this trial. ☐

11. I agree to take part in the above study. ☐

<u>Optional</u>

12. I am willing to be contacted by a research assistant to discuss completion of the diary as explained in the participant information sheet. ☐

13. I am willing to provide a urine sample for further laboratory testing to see if there are active ingredients of the study medication. ☐

For the purposes of the Data Protection Act 1998 the data controller is the University of Southampton and any inquiries relating to your personal information may be addressed to the Southampton Clinical Trials Unit, MP131, Southampton General Hospital, Southampton, SO16 6YD.

___________________________	______________________	____________________
Name of Patient	Signature	Date

___________________________	______________________	____________________
Name of Person taking consent	Signature	Date

When completed:
1 (original) signed consent form to be kept in researcher site file
1 copy for the participant
1 copy to be kept in the medical notes
