# Supplementary material for: Uva-ursi extract and ibuprofen as alternative treatments of adult female urinary tract infection (ATAFUTI): study protocol for a randomised controlled trial
Source: Trials. 2017 Sep 8;18:421. doi: 10.1186/s13063-017-2145-7 (PMC5591533; doi:10.1186/s13063-017-2145-7)
Supplement: Supplementary file 6 — Participant diary. (PDF 515 kb) [file 13063_2017_2145_MOESM6_ESM.pdf]

UNIVERSITY OF Southampton

ATAFUTI

A Trial Investigating Alternative Treatments of Adult Female Urinary Tract Infection.

**Date of Registration**	D	D	M	M	M	Y	Y	Y	Y

**Participant ID**						

**Patient Initials**			

Participant Diary

**CRF**	**0**	**3**

INSTRUCTIONS FOR COMPLETING THE DIARY

We would like you to complete this two week diary. It is divided into 3 sections as below:

Section 1: Please complete this section **on the day** you saw your doctor or Nurse

Section 2: Please complete this section **every day over the next two weeks or until symptoms subside and no further treatments are being used.**

Section 3: Please complete this section **once you have finished entering into Section 2.**

Once you have completed this diary, please return to the address below using the pre-paid addressed envelope provided. When we have received your diary you will receive a £5 voucher from the ATAFUTI team as a thank you.

FREEPOST RTHT-TBHY-ZJJR
ATAFUTI Trial
Southampton Clinical Trials Unit
MP 131
Southampton General Hospital
Tremona Road
Southampton
Hampshire
SO17 1YN

UKCRC Registered Clinical Trials Units

NHS National Institute for Health Research

ATAFUTI

Participant ID						

Participant Initials			

SECTION 1: ABOUT YOU

Please complete this section on the day you saw your doctor.

Part A. Month and year of birth

M	M	M	Y	Y	Y	Y

Part B. History of Urine Infections

1. Have you ever had a urine infection diagnosed by a doctor, at any point in the past not counting this current episode? ***Please tick one box***

Yes ☐ **No** ☐ **Do not know** ☐

If Yes, please complete remaining questions in Part B

***If No or Do not know then go to* Part C**

2. How many times have you been treated for a urine infection in the past 2 years? ***Please tick one box.***

**0**	☐
**1**	☐
**2**	☐
**3 or more**	☐
**Unable to remember**	☐

3. Have you had a urine infection in the last year? **Yes** ☐ **No** ☐

If Yes, how many months since your last one? ☐

UKCRC Registered Clinical Trials Units

NHS National Institute for Health Research

4. How was your last urine infection treated? ***Please tick relevant boxes.***

**Antibiotics**	☐	
**Name of Antibiotic (if known)**		
**Other**	☐	
**If Other, please specify**		
**No Treatment**	☐	
**Do not remember**	☐	

Now please complete Part C

Part C Additional information

1. When you contacted your GP today were you expecting to receive any of the following? ***Please tick one box for each option.***

**Advice**	**Yes** ☐	**No** ☐	**Unsure** ☐
**Tests/Investigations**	**Yes** ☐	**No** ☐	**Unsure** ☐
**Antibiotics**	**Yes** ☐	**No** ☐	**Unsure** ☐
**Other**	**Yes** ☐	**No** ☐	**Unsure** ☐
**If Other, please specify**			

2. Do you believe the following statement?
Please tick one box.

**Herbs might help my symptoms.**	**Yes** ☐	**No** ☐	**Unsure** ☐

ATAFUTI

Participant ID								Participant Initials			

3. Before you went to see your doctor did you try and manage your urine infection with any of the following? ***Please tick all that apply.***

**Cranberry juice**	☐
**Other fruit juice**	☐
**Bicarbonate solution**	☐
**Potassium citrate**	☐
**Other e.g. paracetamol**	☐

If Other, please specify

UKCRC Registered Clinical Trials Units

NHS National Institute for Health Research

Participant ID								Participant Initials			

SECTION 2:
DIARY OF URINE INFECTION SYMPTOMS/PROBLEMS/TREATMENTS

Please fill in the diary on the next few pages to record your symptoms and any treatments (study medication, antibiotics, other medications or products) used. Please start **THIS EVENING** (the evening of the day on which you saw your doctor) and continue to fill this in each evening for 2 weeks or until all symptoms have subsided and no treatments are being taken. **Once you have stopped completing the diary**, please enter the date in the relevant box. If you stopped filling the diary in Week 1 there is no need to complete Week 2. Once your symptoms have settled or after 14 days when you are no longer entering information in Section 2 then please fill in **Section 3**.

For each week the diary is split into two sections – please could you record your symptoms in the first section and all treatments taken in the second section.

ATAFUTI

Participant ID								Participant Initials			

WEEK 1 SYMPTOMS

For your symptoms, the answer you give should reflect how you have felt over the last 24 hours. If you have no symptoms or problems, please enter 0 (to indicate normal/not affected). Equally, if a symptom or problem ends during the period of the diary, enter 0 until the end of the diary.

For each symptom/problem, rate how bad it has been using the following scale. The first shaded column is completed as an example - please fill in your own numbers:

0 = Normal/not affected

1 = Very little problem

2 = Slight problem

3 = Moderately bad

4 = Bad

5 = Very bad

6 = As bad as it could be

**DAY**	**e.g.**	**1**	**2**	**3**	**4**	**5**	**6**	**7**
**DAY OF WEEK**	Mon							
**Symptom/Problem**								
**Fever**	0							
**Pain in the side**	1							
**Blood in urine**	0							
**Smelly urine**	5							
**Burning (Burning or pain when passing urine)**	2							
**Urgency (Having to go in a hurry)**	2							
**Day time frequency (Having to go more often than usual during the day)**	2							
**Night time frequency (Having to go more often than usual during the night)**	0							
**Tummy pain (When not passing urine)**	0							
**Restricted activities**	1							
**Unwell**	0							

ATAFUTI

Participant ID						

Participant Initials			

WEEK 1 TREATMENTS

***For study treatments*: Please enter the number of times you have taken the study medication on each day. (Please note that you only need to record the number of times and not the number of capsules taken).** When you stop taking the study medication, please record the reason for doing this in the relevant table below.

***For other treatments*,** please record details of the treatment taken for your urinary tract infection, either antibiotics or another product (such as cranberry juice or another fruit juice, bicarbonate solution, potassium citrate, ibuprofen, paracetamol) in the table below and enter the number of times you have taken each treatment on that day.

		DAY	e.g.	1	2	3	4	5	6	7
		DAY OF WEEK	Mon							
**Treatments**										
**STUDY MEDICATION**	**Green Capsules (No. of times taken during the day)**		2							
**ANTIBIOTICS**										
**Name of Antibiotic**		**Dose**								
			3							
**OTHER e.g. paracetamol/ibuprofen/fruit juice etc.**										
**Name of Other Medication or Product**		**Strength (if applicable)**								
			1							
			3							
			1							
			1							

Why did you stop taking the study medication? ***Please tick one box.***

Symptoms resolved ☐ **Started taking antibiotics** ☐ **Other** ☐

All study medication taken ☐ **Side effects experienced** ☐

If Other, please specify ____________________

When you stop filling in the diary, i.e. you are no longer experiencing symptoms or taking treatments, please confirm the date here and then complete Section 3.

D	D	M	M	M	Y	Y	Y	Y

If you still have symptoms please continue with week 2

ATAFUTI

Participant ID							Participant Initials			

WEEK 2 SYMPTOMS

***For your symptoms**,* the answer you give should reflect how you have felt over the last 24 hours. If you have no symptoms or problems, please enter 0 (to indicate normal/not affected). Equally, if a symptom or problem ends during the period of the diary, enter 0 until the end of the diary.

For each symptom/problem, rate how bad it has been using the following scale. The first shaded column is completed as an example - please fill in your own numbers:

0 = Normal/not affected

1 = Very little problem

2 = Slight problem

3 = Moderately bad

4 = Bad

5 = Very bad

6 = As bad as it could be

DAY	e.g.	1	2	3	4	5	6	7
DAY OF WEEK	Mon							
**Symptom/Problem**								
**Fever**	0							
**Pain in the side**	1							
**Blood in urine**	0							
**Smelly urine**	5							
**Burning (Burning or pain when passing urine)**	2							
**Urgency (Having to go in a hurry)**	2							
**Day time frequency (Having to go more often than usual during the day)**	2							
**Night time frequency (Having to go more often than usual during the night)**	0							
**Tummy pain (When not passing urine)**	0							
**Restricted activities**	1							
**Unwell**	0							

UKCRC Registered Clinical Trials Units

NHS National Institute for Health Research

ATAFUTI

Participant ID						

Participant Initials			

WEEK 2 TREATMENTS

***For study treatments*: it is unlikely that you will be still be taking the study medication in Week 2 but if you are please enter the number of times you have taken the study medication on each day. (Please note that you only need to record the number of times and not the number of capsules taken).** When you stop taking the study medication, please record the reason for doing this in the relevant table below.

For other treatments, please record details of the treatment taken for your urinary tract infection, either antibiotics or another product (such as cranberry juice or another fruit juice, bicarbonate solution, potassium citrate, ibuprofen, paracetamol) in the table below and enter the number of times you have taken each treatment on that day.

		DAY	e.g.	1	2	3	4	5	6	7
		DAY OF WEEK	Mon							
Treatments										
STUDY MEDICATION	Green Capsules (No. of times taken during the day)		2							
ANTIBIOTICS										
Name of Antibiotic		Dose								
			3							
OTHER e.g. paracetamol/ibuprofen/fruit juice etc.										
Name of Other Medication or Product		Strength (if applicable)								
			3							
			1							
			1							
			2							

Why did you stop taking the study medication? ***Please tick one box.***

**Symptoms resolved**	☐	**Started taking antibiotics**	☐	**Other**	☐
**All study medication taken**	☐	**Side effects experienced**	☐		
**If Other, please specify**					

When you stop filling in the diary, i.e. you are no longer experiencing symptoms or taking any treatments and stop filling in the diary, please confirm the date here.

D	D	M	M	M	Y	Y	Y	Y

Now please complete Section 3.

UKCRC Registered Clinical Trials Units

NHS National Institute for Health Research

ATAFUTI

Participant ID						

Participant Initials			

Section 3:
These questions are to be filled in when your symptoms have settled or after 14 days

Date completed D D M M M Y Y Y Y

Since you saw your doctor:

1. Have you consulted with a health professional from your **general practice** or **out of hour's provider** about your urine infection? (*Do not count the original visit*)

Yes ☐ **No** ☐

If YES then who did you see? (If you did not see the person in question then please enter 0)

	**How many times?**		
**GP at Surgery**			
**Nurse at Surgery**			
**GP at home**			
**Out of hour's doctor**			
**Other**		**Please specify**	

2. Have you consulted with a health professional in an **accident and emergency** department about your urine infection?

Yes ☐ **No** ☐ **If YES, then how many times?** ☐

3. Have you been seen by a specialist (not including an admission to hospital) about your urine infection? This might occur if your GP or another healthcare professional had referred you for an urgent opinion about your urine infection, but you were not admitted to hospital).

Yes ☐ **No** ☐ **If YES, then how many times?** ☐

ATAFUTI

Participant ID								Participant Initials			

4. Have you been admitted to hospital for a problem related to your urine infection?

Yes ☐ **No** ☐

If YES, then how many nights did you spend in hospital? ☐

5. As a result of the help managing your symptoms in this study and the advice you were given, do you feel you are:

**Able to cope with life?**	**Much better**	☐	**Better**	☐	**Same or less**	☐
**Able to understand your illness?**	**Much better**	☐	**Better**	☐	**Same or less**	☐
**Able to cope with your illness?**	**Much better**	☐	**Better**	☐	**Same or less**	☐
**Able to keep yourself healthy?**	**Much better**	☐	**Better**	☐	**Same or less**	☐
**Confident about your health?**	**Much more**	☐	**Better**	☐	**Same or less**	☐
**Able to help yourself?**	**Much more**	☐	**Better**	☐	**Same or less**	☐

6. Do you think you that took real Uva Ursi? ***Please tick one box.***

Yes ☐ **No** ☐ **Unsure** ☐

7. Do you think that the study medication helped your symptoms? ***Please tick one box.***

Yes ☐ **No** ☐ **Unsure** ☐

8. Do you think that any additional treatment that you took helped your symptoms? ***Please tick one box.***

Yes ☐ **No** ☐ **Unsure** ☐

If YES, please specify. ☐

Participant ID						

Participant Initials			

That is the end of the questions!

The information you have provided will remain confidential and the pooled data will help us to improve our management and treatment of patients with urine infections.

Please add any comments you have about urine infections or this study.

Once we have received your diary we will send you a £5 voucher to say thank you very much for completing the symptom diary and questions. ***Please remember to return any unused trial medication in the prepaid addressed packaging that you were given by your GP or Nurse.***

If you have any problems or queries, please contact:

Catherine Simpson
ATAFUTI Clinical Trials Coordinators
Southampton Clinical Trials Unit
MP 131
Southampton General Hospital
Tremona Road
Southampton
Hampshire
SO16 6YD
023 8120 5171

THANK YOU!

You have made a valuable contribution to this important medical research.

UKCRC Registered Clinical Trials Units

NHS National Institute for Health Research
