# Supplementary material for: Uva-ursi extract and ibuprofen as alternative treatments of adult female urinary tract infection (ATAFUTI): study protocol for a randomised controlled trial
Source: Trials. 2017 Sep 8;18:421. doi: 10.1186/s13063-017-2145-7 (PMC5591533; doi:10.1186/s13063-017-2145-7)
Supplement: Supplementary file 7 — Qualitative Research Patient Information Sheet. (PDF 195 kb) [file 13063_2017_2145_MOESM7_ESM.pdf]

UNIVERSITY OF OXFORD
NUFFIELD DEPARTMENT OF PRIMARY CARE HEALTH SCIENCES
University of BRISTOL
UNIVERSITY OF Southampton
SOUTHAMPTON CLINICAL TRIALS UNIT

Participant Information Sheet

Patient Interview

ATAFUTI

A Trial Investigating Alternative Treatments for Adult Female Urinary Tract Infection.

Version number v3 17/03/15

Ethics number: 14/SC/1143

We would like to invite you to participate in an interview as part of a research study looking into alternative treatments for urinary tract infections. The interviews are being undertaken as part of a PhD Thesis at the University of Southampton. Before you decide whether or not to take part, we would like you to understand why the research is being carried out and what it will involve. Please take time to read the following information carefully.

Please ask us if there is anything that is not clear or if you would like more information.

Part 1

What is the purpose of the study?
Urinary Tract Infections are very common, and alternative treatments to antibiotics are being sought to relieve the symptoms. We want to learn about your experience of urinary tract infections, and are interested in your views about being offered an alternative treatment such as a herbal medicine.   If you recently agreed to take part in the trial to test these treatments we would also like to find out how you felt about being asked to delay taking antibiotics to relieve your symptoms.

Why have I been invited?
You have been chosen because you have recently been diagnosed by your GP with a urinary tract infection.

Do I have to take part?
No. It is up to you to decide whether or not to take part. A decision not to take part, will not affect the standard of care you receive.
What will happen to me if I agree to take part?

NHS National Institute for Health Research

After carefully reading this information sheet and asking any questions you may have about the study you will be asked to provide the researcher with your contact details by completing the reply slip at the bottom of the accompanying letter and returning this using the prepaid addressed envelope enclosed. The researcher will contact you at a later date, but within three months of your participation in the trial (by email/letter/telephone) to see if you are still willing to take part. You will then be sent a Consent Form for the in-depth interview, which you will be asked to sign and return to the PhD Researcher. The consent form will confirm that you have read and understood the information in this document.

If you agree to take part in the informal interview, you will be contacted by the PhD Researcher by phone/email to arrange a convenient time. The informal interview will last 30-60 minutes and be conducted in your home unless you would prefer to come to the GP practice instead or be interviewed over the telephone. The interview will be conducted by a female PhD Researcher. You will be asked questions about your Urinary Tract Infection symptoms and any treatment you have received. You will also be asked about your views on antibiotics, as well as on an alternative treatment such as herbal medicine.

The interview will be audio recorded so that the researchers can write it up and study it at a later day. Your name is not used in this process and any quotes from the interview will remain anonymous.

What are the other possible disadvantages and risks of taking part?
You will be giving up some of your time to attend the interview. Slightly embarrassing issues (i.e. urinary tract infections) will be talked about.

What are the possible benefits of taking part?
Your participation may help to give important information about how best to treat people with Urinary Tract Infection in the future.

This completes Part 1 of the Information Sheet.
If the information in Part 1 has interested you and you are considering participation, please read the additional information in Part 2 before making any decision.

Part 2

What will happen if I don't want to carry on with the study?
You can withdraw from the study at any time you don't have to give a reason. The standard of your care will not be affected. Information collected up to the time you withdraw may still be used.

What if there is a problem?
If you have a concern about any aspect of this study, you should speak to the researcher Tel: 07935 280202 who will do their best to answer your questions or contact the trial co-ordinator – contact details below.

If you remain unhappy and wish to complain formally, you can do this through the normal National Health Service complaints Procedure. Details can be obtained through the following NHS web site.
http://www.nhs.uk/choiceintheNHS/Rightsandpledges/complaints/Pages/AboutNHScomplaints.aspx

Will my taking part in this study be kept confidential?
All information which is collected about you during the course of the research will be kept securely, anonymously and strictly confidential. Your name will not be used in the collection, storage or publication of any research material.

What will happen to the results of the research study?
The findings from this study will be written up as part of a PhD thesis after the study data has been analysed (approximately during 2016). No identifiable information will be included which could compromise the confidentiality of the study participants. The findings may also be written up and published in a medical journal. Any participant who wishes to obtain a copy of the publication should contact the Southampton Clinical Trials Unit, MP131, Southampton General Hospital, Tremona Road, Southampton, Hants, SO16 6YD.

If results are conclusive they may be used to influence future NHS guidelines for treatment of Urinary Tract Infection.

Who is organising and funding the research?
This study has been organised by the Universities of Southampton, Oxford and Bristol and the trial is being run by the Southampton Clinical Trials Unit. The study is funded by a grant from the National Institute for Health Research, National School for Primary Care Research. The study Sponsor is the University of Southampton.

Who has reviewed the study?
All research in the NHS is looked at by independent group of people, called a Research Ethics Committee, to protect your interests. This study has been reviewed by NRES Committee South Central – Hampshire A.

Further Information and contact details
If you have any questions, please contact:

PhD Researcher, Jeanne Trill

Tel: Tel: 07935 280202
Email: Jeanne.Trill@btinternet.com

Or Trial Co-ordinator, Catherine Simpson at the Southampton Clinical Trials Unit,

Tel: 023 8120 5171
Email: CTU@soton.ac.uk

If you have any further questions about your illness, please discuss them with your GP.

For further information about Urinary Tract Infection you may also find it helpful to look at the following NHS internet link:
http://www.nhs.uk/conditions/Urinary-tract-infection-adults/Pages/Introduction.aspx

Thank you for taking the time to read this information sheet.
