# Supplementary material for: Uva-ursi extract and ibuprofen as alternative treatments of adult female urinary tract infection (ATAFUTI): study protocol for a randomised controlled trial
Source: Trials. 2017 Sep 8;18:421. doi: 10.1186/s13063-017-2145-7 (PMC5591533; doi:10.1186/s13063-017-2145-7)
Supplement: Supplementary file 8 — Qualitative Research Patient Informed Consent. (PDF 369 kb) [file 13063_2017_2145_MOESM8_ESM.pdf]

(TO BE PRINTED ON LOCAL HEADED PAPER)

Ethics number: 14/SC/1143

Centre ID: ☐☐

Participant's Initials: ☐☐☐ (If no middle initial insert '-')

Participant's Month & Year of Birth: M M M Y Y Y Y

Participant Trial ID(if applicable): ☐☐☐☐☐☐

ATAFUTI

A Trial Investigating Alternative Treatments of Adult Female Urinary Tract Infection.

CONSENT FORM – PATIENT INTERVIEW

Name of Researcher: Professor Michael Moore

The informed consent consists of two parts – the information sheet and this consent form.

Please Initial Box

1. I confirm that I have read and understand the participant information sheet (version 3 dated 17/03/15) for the above study. I have had the opportunity to consider the information, ask questions and have had these answered satisfactorily. ☐

2. I understand that my participation in the interview is voluntary and that I am free to withdraw at any time without giving any reason, and without my medical care or legal rights being affected. ☐

3 I consent to the collection and use of information about me in accordance with the participant information sheet. ☐

4 I give permission for my interview to be audio recorded. I understand that my name will not be used in this process and that any quotes used will remain anonymous. ☐

UKCRC Registered Clinical Trials Units

NHS National Institute for Health Research

5 I understand that any data collected, at the interview, up to the time of my withdrawal may continue to be used in the above study. ☐

6 I agree to take part in the interview for the above study. ☐

For the purposes of the Data Protection Act 1998 the data controller is the University of Southampton and any inquiries relating to your personal information may be addressed to the Southampton Clinical Trials Unit, MP131, Southampton General Hospital, Southampton, SO16 6YD.

____________________	____________________	____________________
Name of Patient	Signature	Date
____________________	____________________	____________________
Name of Person taking consent	Signature	Date

When completed:
1 (original) signed consent form to be kept in Medical Notes
1 copy for the participant
1 copy for the researcher site file

UKCRC Registered Clinical Trials Units
NHS National Institute for Health Research
