# Supplementary material for: Uva-ursi extract and ibuprofen as alternative treatments of adult female urinary tract infection (ATAFUTI): study protocol for a randomised controlled trial
Source: Trials. 2017 Sep 8;18:421. doi: 10.1186/s13063-017-2145-7 (PMC5591533; doi:10.1186/s13063-017-2145-7)
Supplement: Supplementary file 9 — Qualitative Research GP Information Sheet. (PDF 250 kb) [file 13063_2017_2145_MOESM9_ESM.pdf]

UNIVERSITY OF OXFORD
NUFFIELD DEPARTMENT OF PRIMARY CARE HEALTH SCIENCES
University of BRISTOL
UNIVERSITY OF Southampton
SOUTHAMPTON CLINICAL TRIALS UNIT

GP Participant Information Sheet

GP Interview

ATAFUTI

Alternative Treatments of Adult Female Urinary Tract Infection: a double blind, placebo controlled, factorial randomised trial of uva ursi and open pragmatic trial of ibuprofen

Version number 3 17-03-15

Ethics number: 14/SC/1143

We would like to invite you to take part in an interview for qualitative part of the above research study. This is being undertaken as part of a PhD Thesis at the University of Southampton. Before you decide whether or not to take part, we would like you to understand why the research is being done and what it will involve. Please take time to read the following information carefully.

Please ask us if there is anything that is not clear or if you would like more information.

Part 1

What is the purpose of the study?

Urinary Tract Infections are very common, and alternative treatments to antibiotics are being sought to relieve the symptoms. We want to learn about your experience of treating urinary tract infections, and are interested in your views about alternative treatments such as ibuprofen and/or herbal medicine. If your patients recently took part in the trial to test these treatments we would also like to find out how you felt about being asked to delay prescribing antibiotics to relieve the symptoms.

Why have I been invited?

You have been chosen because your practice has been involved in the clinical trial.

Do I have to take part?

No. It is up to you to decide whether or not to take part. You are free to withdraw at any time and without giving a reason.

What will happen to me if I agree to take part?

After carefully reading this information sheet and asking the researcher any questions you may have about the study you will be asked to sign a consent form. The consent form will confirm that you have read and understood the information in this document.

If you agree, you will be asked to take part in an informal interview and will be contacted by the PhD Researcher by phone/email to arrange a convenient time. The informal interview will last 15-30 minutes and be conducted in your surgery by a PhD Researcher. You will be asked questions about your treatment of Urinary Tract Infections. You will also be asked about your views on antibiotics, as well as on an alternative treatment such as herbal medicine.

The interview will be audio recorded so that the researchers can write it up and study it at a later day. Your name is not used in this process and any quotes from the interview will remain anonymous.

What is the treatment that is being tested?
This trial is looking at a herbal medicine that has possible beneficial effects for treating Urinary Tract Infection. The active ingredient 'Arbutin' which has antiseptic and anti-inflammatory properties is extracted from Uva-Ursi leaves. We are looking at the effectiveness of taking the herb for relieving Urinary Tract Infection symptoms. In addition, we are also investigating whether advice given to patients by their GP or nurse to take ibuprofen could also help relieve symptoms and reduce the use of antibiotics.

What are the other possible disadvantages and risks of taking part?
You will be giving up some of your time to attend.

What are the possible benefits of taking part?
Your participation may help to give important information about how best to treat people with Urinary Tract Infection in the future.

This completes Part 1 of the Information Sheet.
If the information in Part 1 has interested you and you are considering participation, please read the additional information in Part 2 before making any decision.

Part 2

What will happen if I don't want to carry on with the study?
You can withdraw from the study at any time and you don't have to give a reason. Information collected at the interview up to the time you withdraw may still be used.

What if there is a problem?
If you have a concern about any aspect of this study, you should ask to speak with the researcher (tel no to be entered here who will do their best to answer your questions or the Trial Coordinator - contact details below).

Will my taking part in this study be kept confidential?
All information which is collected about you during the course of the interview will be kept strictly confidential. Your name will not be used in the collection, storage or publication of any research material.

What will happen to the results of the research study?
The findings from this study will be written up as part of a PhD thesis after the study data has been analysed (approximately during 2016). No identifiable information will be included which could compromise the confidentiality of the study participants. The findings may also be written up and published in a medical journal. Any participant who wishes to obtain a copy of the publication should contact the University of Southampton Clinical Trials Unit, MP131, Southampton General Hospital, Tremona Road, Southampton, Hants, SO16 6YD.

If results are conclusive they may be used to influence future NHS guidelines for treatment of Urinary Tract Infection.

Who is organising and funding the research?
This study has been organised by the Universities of Southampton, Oxford and Bristol and the trial is being run by the Southampton Clinical Trials Unit. The study is funded by a grant from the National Institute for Health Research, Health Technology National School for Primary Care Research. The study Sponsor is the University of Southampton.

Who has reviewed the study?
All research in the NHS is looked at by independent group of people, called a Research Ethics Committee, to protect your interests. This study has been reviewed by NRES Committee South Central – Hampshire A.

Further Information and contact details
If you have any questions, please contact:

PhD Researcher, Jeanne Trill

Tel: 07935 280202
Email: Jeanne.Trill@btinternet.com

Or **Trial Co-ordinator**, Catherine Simpson at the Southampton Clinical Trials Unit,

Tel: 023 8120 5171
Email: CTU@soton.ac.uk

Thank you for taking the time to read this information sheet.
