# Supplementary material for: Uva-ursi extract and ibuprofen as alternative treatments of adult female urinary tract infection (ATAFUTI): study protocol for a randomised controlled trial
Source: Trials. 2017 Sep 8;18:421. doi: 10.1186/s13063-017-2145-7 (PMC5591533; doi:10.1186/s13063-017-2145-7)
Supplement: Supplementary file 10 — Qualitative Research GP Informed Consent. (PDF 310 kb) [file 13063_2017_2145_MOESM10_ESM.pdf]

Ethics number: 14/SC/1143

Centre ID: | | |

Participant's Initials: | | | | (If no middle initial insert '-')

Participant Trial ID: | | | | | | (Centre ID + GP's Initials)

ATAFUTI

Alternative Treatments of Adult Female Urinary Tract Infection: a double blind, placebo controlled, factorial randomised trial of Uva ursi and open pragmatic trial of ibuprofen

GP CONSENT FORM – GP INTERVIEW

Name of Researcher: Professor Michael Moore

The informed consent consists of two parts – the information sheet and this consent form.

Please Initial Box

1. I confirm that I have read and understand the participant information sheet (version 3 dated 17/03/15) for the above study. I have had the opportunity to consider the information, ask questions and have had these answered satisfactorily. ☐

2. I understand that my participation is voluntary and that I am free to withdraw at any time without giving any reason, and without my legal rights being affected. ☐

3. I consent to the collection and use of information about me from the interview in accordance with the participant information sheet. ☐

4. I give permission for my interview with the researcher to be audio recorded. I understand that my name will not be used in this process and that any quotes used will remain anonymous. ☐

5. I understand that any data collected from me up to the time of my withdrawal may continue to be used in the above study. ☐

6. I agree to take part in the interviews for the above study. ☐

For the purposes of the Data Protection Act 1998 the data controller is the University of Southampton and any inquiries relating to your personal information may be addressed to the Southampton Clinical Trials Unit, MP131, Southampton General Hospital, Southampton, SO16 6YD.

Name of Participant	Signature	Date
Name of Person taking consent	Signature	Date

When completed:
1 (original) signed consent form to be kept in Medical Notes
1 copy for the participant
1 copy for the researcher site file
