# Supplementary material for: Uva-ursi extract and ibuprofen as alternative treatments of adult female urinary tract infection (ATAFUTI): study protocol for a randomised controlled trial
Source: Trials. 2017 Sep 8;18:421. doi: 10.1186/s13063-017-2145-7 (PMC5591533; doi:10.1186/s13063-017-2145-7)
Supplement: Supplementary file 11 — Trial amendments. (PDF 114 kb) [file 13063_2017_2145_MOESM11_ESM.pdf]

V1<br>25/6/14	Amendment 1 in ERGO = amendments to initial Ethics application following provisional opinion from REC. To keep nomenclature consistent the first REC Substantial amendment has been called AM 02 inline with ERGO
V2<br>12/11/14	Protocol amended for administrative and clarification purposes. Protocol v2<br>Informed Consent Forms for the Main Trial amended to include consent to have a pregnancy test if required and to provide a urine sample for microbial culture.<br>Patient Information Sheets for the Main Trial amended to clarify how participant contact details will be securely stored.<br>Trial Participant Treatment Card amended to include telephone number for the Southampton Clinical Trials Unit.<br>Qualitative Research GP Invitation Letter – this is a new document.<br>Symptom Diary by Recall – this is a new document.<br>Symptom Diary by Recall Guidelines – this is a new document.
V3<br>17/03/15	Modified trial design replacing ibuprofen/placebo with 'advice to take ibuprofen/no advice'. This resulted in changes to the trial titles (full and simplified); primary and secondary objectives; the method of statistical analysis and sample size.<br>Patient Pack Instruction Cards have been designed to be included in the patient packs to instruct the clinician to advise the participant to take ibuprofen or to give no recommendation.<br>The IMP labels have been amended in line with the new trial design.<br>The Investigator Brochure for Uva ursi has been changed to remove all reference to ibuprofen and NuPharm laboratories and to document that Essential Nutrition will be responsible for packaging, labelling and distribution of the Patient Packs.<br>The IMPDs for Uva ursi and placebo have been updated to include the 6 and 9 month stability data and the proposed shelf life for Uva ursi capsules is 21 months based on the 9 month real-time stability data.<br>The protocol and study documents have been amended in line with the new trial design and all references to ibuprofen have been removed from patient facing documents. Amended study documents include: ICFs (4), PISs (4), GP notification letter, Trial Participant Treatment Card, Clinic Poster, Qualitative Research Invitation Letters (2), Participant Diary.<br>The participant focus group discusions have been removed.<br>The recording of AR/AEs restricted to those judged to be possibly related to the study - medical areas/symptoms specified.<br>Participant Urine Collection Instruction Sheet - this is a new document.<br>Some minor changes to the text of the protocol and trial documents for clarification purposes or to correct typographical and grammatical errors. New logos for SCTU and the trial added where appropriate.<br>Changes to the DMEC membership.

V4<br>15/05/15	Version 3 of the protocol stated incorrectly in the Trial Synopsis that Uva ursi should be taken 4 times a day instead of 3 times a day. In the main body of the protocol the dosage regimen is specified correctly. This was a typographical error but as the Trial Synopsis is the first point of reference to the IMP in the protocol it was considered a significant error.3. Patient Information Sheet for the Main Trial amended toa) clarify why a pregnancy test is being carried out.b) include that if participant falls pregnant whilst taking the IMP they will need to be followed up by their GP until the outcome of their pregnancy is knownc) correct grammatical error - Uva Ursi changed to Uva ursi through out4. Patient Information Sheet for the Main Trial + Day 4 Urine Collection amended as above for PIS for the Main Trial.5. Participant Diary amended by merging the treatment tables on p7 & p9 to collect information more accurately as to when participants took any other treatments. The same data is being collected but in a different format. Some minor changes have been made to the text and order of the diary to correct typographical and grammatical errors or for clarification purposes.
V5<br>24/03/16	IMPDs updated with 18 month stability data to give shelf life of 30 months. Increased sample size from 328 to 376 and extended recruitment period to 30/09/16. Protocol amended to clarify that GPs and nurse prescribers (NP) can be interviewed as part of the qualitative research study; to clarify that these interviews can take place over the phone; to specify the number of GP/NPs that will be interviewed and to clarify the consent process for both patient and GP/NP interviews. In addition a few minor changes have been made to the protocol for clarification purposes. PIS updated to include explanation why participants should consult their GP immediately if they develop symptoms of a kidney infection and to clarify that they will be contacted by the research team if after 3 weeks they have not returned their diary or there is key information missing. Participant Diary amended to explain that participants will receive a £5 voucher when they have returned a fully completed diary. The list of staff conducting specified research procedures has been extended to include HCAs and CTAs.
V6<br>13/06/16	Resubmitted as for AM07 with V6 protocol. Exclusion criterion "Recruited to another interventional randomised control trial in previous 4 weeks" amended to " Recruited to another interventional trial in previous 6 weeks" as requested by the MHRA.
