# Supplementary material for: Uva-ursi extract and ibuprofen as alternative treatments of adult female urinary tract infection (ATAFUTI): study protocol for a randomised controlled trial
Source: Trials. 2017 Sep 8;18:421. doi: 10.1186/s13063-017-2145-7 (PMC5591533; doi:10.1186/s13063-017-2145-7)
Supplement: Supplementary file 12 — CSP NHS permissions for ATAFUTI. (PDF 107 kb) [file 13063_2017_2145_MOESM12_ESM.pdf]

Region-wide NHS permissions were given for the ATAFUTI Trial (CSP 115144) by the following bodies:

CRN Wessex on behalf of all independent contractors within the CRN Wessex area.

CRN Thames Valley and South Midlands on behalf of independent contractors in the following NHS Primary Care areas

- Formerly Oxfordshire PCT
- Formerly Buckinghamshire PCT
- Formerly Berkshire East PCT
- Formerly Berkshire West PCT
- Formerly Milton Keynes PCT

RM&G Consortium for Kent & Medway on behalf of independent primary care providers in

- Kent & Medway,
- Kent Community Health NHS Trust,
- Medway Community Healthcare CIC,
- Kent & Medway NHS & Social Care Partnership Trust
- South East Coast Ambulance NHS Trust

Sussex NHS Research Consortium on behalf of GP Practices in

- Formerly Surrey PCT.
- Formerly Brighton & Hove City PCT
- Formerly East Sussex Downs and Weald PCT
- Formerly Hastings & Rother PCT
- Formerly West Sussex PCT.

Research Management Governance Team hosted by NHS Cambridgeshire & Peterborough CCG on behalf of GP practices in CRN Eastern

Bath R&D on behalf of GP practices in

- NHS Bath & NE Somerset CCG
- NHS Swindon CCG
- NHS Wiltshire CCG

Royal Devon & Exeter Shared Research Management Service on behalf of primary care providers in

- Formerly NHS Devon
- Formerly NHS Plymouth
- Formerly NHS Cornwall & Isle of Scilly
- Formerly Torbay Care Trust

Avon Primary Care Research Collaborative on behalf of primary care providers in

- NHS Bristol CCG
- North Somerset CCG
- South Gloucestershire CCG

Gloucestershire Research Support Services on behalf of primary care providers in Gloucestershire CCG.
